# A methodology review on the incremental prognostic value of computed tomography biomarkers in addition to Framingham risk score in predicting cardiovascular disease: the use of association, discrimination and reclassification

**DOI:** 10.1186/s12872-018-0777-5

**Published:** 2018-02-21

**Authors:** Chun Lap Pang, Nicola Pilkington, Yinghui Wei, Jaime Peters, Carl Roobottom, Chris Hyde

**Affiliations:** 1 University of Plymouth, Plymouth University Peninsula Schools of Medicine and Dentistry, John Bull Building, Tamar Science Park, Research Way, Plymouth, PL6 8BU UK; 2 Plymouth Hospitals NHS Trust, Derriford Hospital, Imaging Department, Derriford Rd, Plymouth, PL6 8DH UK; 3 Plymouth Hospitals NHS Trust, Derriford Hospital, Department of Anaesthetics, Derriford Rd, Plymouth, PL6 8DH UK; 4 University of Plymouth, School of Computing, Electronics and Mathematics, Plymouth, PL4 8AA UK; 5 University of Exeter, South Cloisters, St Luke’s Campus, Exeter, EX1 2LU UK; 6 Primary Care Plymouth, Room N9, ITTC Building, Davy Road, Plymouth Science Park, Derriford, Plymouth, Devon PL6 8BX UK

**Keywords:** Prognosis, Framingham risk score: Calcium score, Thoracic calcium score, Computed tomographic coronary angiogram

## Abstract

**Background:**

Computed tomography (CT) biomarkers claim to improve cardiovascular risk stratification. This review focuses on significant differences in incremental measures between adequate and inadequate reporting practise.

**Methods:**

Studies included were those that used Framingham Risk Score as a baseline and described the incremental value of adding calcium score or CT coronary angiogram in predicting cardiovascular risk. Searches of MEDLINE, EMBASE, Web of Science and Cochrane Central were performed with no language restriction.

**Results:**

Thirty five studies consisting of 206,663 patients (me*n* = 118,114, 55.1%) were included. The baseline Framingham Risk Score included the 1998, 2002 and 2008 iterations. Selective reporting, inconsistent reference groupings and thresholds were found. Twelve studies (34.3%) had major and 23 (65.7%) had minor alterations and the respective Δ AUC were significantly different (*p* = 0.015). When the baseline model performed well, the Δ AUC was relatively lower with the addition of a CT biomarker (Spearman coefficient = − 0.46, *p* < 0.0001; *n* = 33; 76 pairs of data). Other factors that influenced AUC performance included exploration of data analysis, calibration, validation, multivariable and AUC documentation (all *p* < 0.05). Most studies (68.7%) that reported categorical NRI (*n* = 16; 46 pairs of data) subjectively drew strong conclusions along with other poor reporting practices. However, no significant difference in values of NRI was found between adequate and inadequate reporting.

**Conclusions:**

The widespread practice of poor reporting particularly association, discrimination, reclassification, calibration and validation undermines the claimed incremental value of CT biomarkers over the Framingham Risk Score alone. Inadequate reporting of discrimination inflates effect estimate, however, that is not necessarily the case for reclassification.

**Electronic supplementary material:**

The online version of this article (10.1186/s12872-018-0777-5) contains supplementary material, which is available to authorized users.

## Key messages

- Selective reporting of association and non-standardisation of cut points make evidence synthesis difficult
- There was a negative correlation between improved discrimination and the baseline performance of Framingham Risk Score
- No evidence was found between poor reporting practice and the magnitude of categorical net reclassification index

## Background

Prediction models are not perfect [1] and often have methodological weaknesses meaning few are used in everyday practice [2]. Framingham Risk Score (FRS) [3] is one of the exceptions because of its extensive validation [1]. It is however, not perfect with researchers attempting to improve prediction within the intermediate category [4]. Numerous other tests have been investigated hoping to improve upon this base model [5]. This is also in keeping with the rising interest in novel biomarkers in medicine [6], particularly in the field of cardiovascular [7] and cancer [8] risk prediction. In search of a surrogate biomarker that detects subclinical disease, coronary calcium score (CACS) has been investigated for more than a decade [9] with some proposing screening with computed tomography (CT) in the general population [10]. Also the quality of both primary and secondary prognostic studies is generally poor [11] but with a few exceptions [6]. Thoracic calcium score is considered a relative of CACS where the Agatston method [12] is applied to the thoracic aorta [13]. CT coronary angiogram (CTCA) has established itself in the acute chest pain setting and is now being investigated as a tool of reclassifying cardiac risk based on luminal stenosis (and other characteristics) in the CONFIRM cohort [14]. These imaging biomarkers generate substantial interest and may add incremental value to traditional Framingham risk factors. Given the known methodological issues [15–17], we looked for differences between adequate and inadequate reporting practice.

## Methods

This article is reported in line with Preferred Reporting Items for Systematic Reviews and Meta-Analyses [18]. The protocol for this review was registered on PROSPERO (2015:CRD42015023795) [19]. CLP searched MEDLINE, EMBASE, Web of Science and the Cochrane Central Register of Controlled Trials in July 2015. The bibliographies of all included studies were searched for further potential studies. Attempts were made to retrieve missing information in the included publications by contacting authors and grey literature was searched [20, 21]. Only full text publications were included. No language restrictions were applied. An update search was carried out in June 2016.

### Screening Process & Study Selection

Two reviewers (CLP, NP) conducted title and abstract, and then full-text screening, against the inclusion criteria. A third reviewer (CH) was involved if disagreements were not resolved by consensus. We only included studies that examined Agatston or CACS, thoracic aorta calcium score (TACS) or CTCA as new predictors and used any iteration of FRS as a baseline model. We included any cohort study that reported the association of FRS with the defined predictors and cardiac endpoints and/or cardiovascular comorbidities. In addition, these studies had to report one of the following: summary statistics indicating incremental value of the predictor of interest in addition to the old model, such as difference in area under the receiver operating characteristics curve (Δ AUC) [22, 23], category-based net reclassification index (NRI) [24, 25], integrated discrimination improvement (IDI) [26], relative IDI (rIDI) [27] or other reclassification measures. Composite endpoints [28] were considered and studies using surrogate outcomes were excluded [29].

### Data extraction

Two authors (CLP, NP) independently extracted data from the included studies, recording the first author, journal, publication year, outcome assessed, population evaluated and their inferences on whether the additional predictor improves prediction beyond the FRS. Publications were classified whenever possible as defined by Framingham/Wilson 1998, Framingham/Adult Treatment Panel III (ATP) 2002 and Framingham/ D’Agnostino 2008 [30, 31]. Original 95% CI, standard error, standard deviation or *p*-values of summary estimates of interest were extracted [32, 33]. If the Kaplan-Meier survival curve was available, any missing hazard ratio was estimated (by YW) [34]. Specifically, the standard of reporting effect sizes that signalled incremental prognostic value was evaluated. The choice of optimal cut points/thresholds was also examined, particularly in relation to size effects that indicate association [35]. Various methods of quantifying incremental prognostic value of an additional test have been described [36]. We focused on the reporting characteristics of multivariable regression, calibration, discrimination and reclassification [15]. For the documentation of multivariable regression, we determined its adequacy based on the availability of information on whether an additional predictor was significant at *p* < 0.05 level, or the use of tests that penalised the inclusion of an additional predictor. For discrimination, we assessed the documentation of the baseline AUC of FRS and the Δ AUC as a result of an additional predictor of interest. The adequacy of reporting baseline AUC relied on accurate documentation of the FRS as originally published [15]. In brief, the calculation of FRS could be threatened by addition, deletion or modification of the original FRS items. Other aspects included whether coronary heart disease (CHD) was measured and the measured population was similar to the original FRS population. For reclassification, all publications were searched for NRI calculation or results. The type of NRI was verified. We considered established categories (e.g. < 10%, 10–20%, > 20%) or any justified use of categories as appropriate to relevant data sets. The recommendation of reporting reclassification was taken from [37].

### Critical appraisal

We rated study quality using the Quality In Prognosis Studies (QUIPS) tool [38]. Two reviewers (CLP, NHP) conducted the quality assessment on the major aspects and two reviewers (JP, YHW) assessed the statistical aspect of the studies independently.

### Data analysis

For studies where the 95% CI or standard error was not reported, a correlation coefficient of 0.3 between FRS and CACS/CTCA was used to allow estimation of the 95% CI for Δ AUC based on data from [39]. Numbers were displayed as exact numbers, median or percentages. The alteration of the risk factors used to calculate FRS was assessed based on previously published items [15] with modifications. The items were scored ordinally as either yes, no or unclear. FRS model of the 1998 iteration was scored against 18 items. FRS model of the 2002 and 2008 iterations were scored against 15 items (3 diabetes related items discounted). The summation of the individual item score indicated the overall level of alteration which was dichotomised into a binary variable: minor and major alterations. The threshold for dichotomisation was based on the median number of items altered among the included studies. The summary of NRIs and AUCs was displayed as medians and interquartile ranges. NRIs and AUCs were subsequently split into two groups depending on the practice of reporting being either adequate or inadequate, or as equivalent binary groups. The aspects of reporting were based on previously published work on AUC [15] and NRI [37] with adaptations. The respective groups were then compared using the Wilcoxon sign rank test at significance level *p* < 0.05. Specifically, we were looking for any particular practice of reporting AUC or NRI leading to excessive claims of any additional predictor. All statistical analysis was carried out using STATA version 14.0 (StataCorp, College Station, Texas).

## Results

### Included studies

**Fig. 1:**
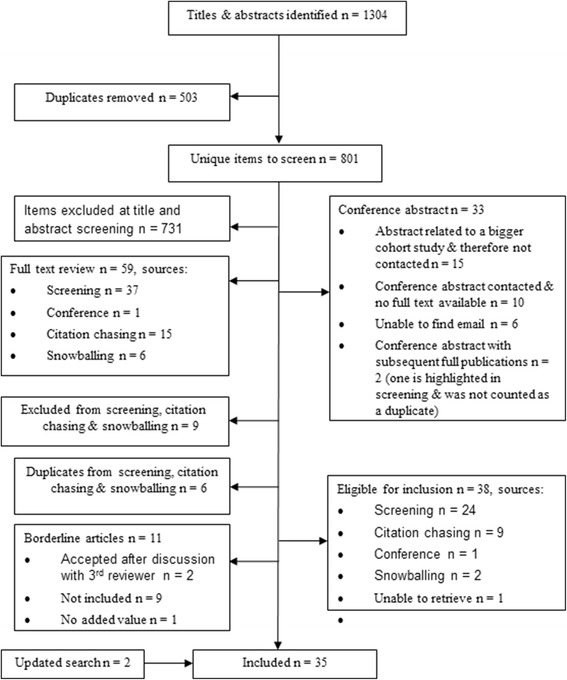
The Preferred Reporting Items for Systematic Reviews and Meta-analysis Flow Diagram

Eight hundred and one unique hits were screened, leading to 35 studies (Fig. 1) encompassing 206,663 patients (men = 118,114, 55.1%) [4, 5, 9, 13, 14, 39–68]. All publications concluded that at least 1 imaging biomarker indicated either independent association with composite endpoints, improved discrimination or classification beyond traditional risk factors. However, there were reservations about TACS [13, 48] and some argued against the reclassification properties of CACS and CTCA [52, 59].

### Quality of included studies

**Fig. 2:**
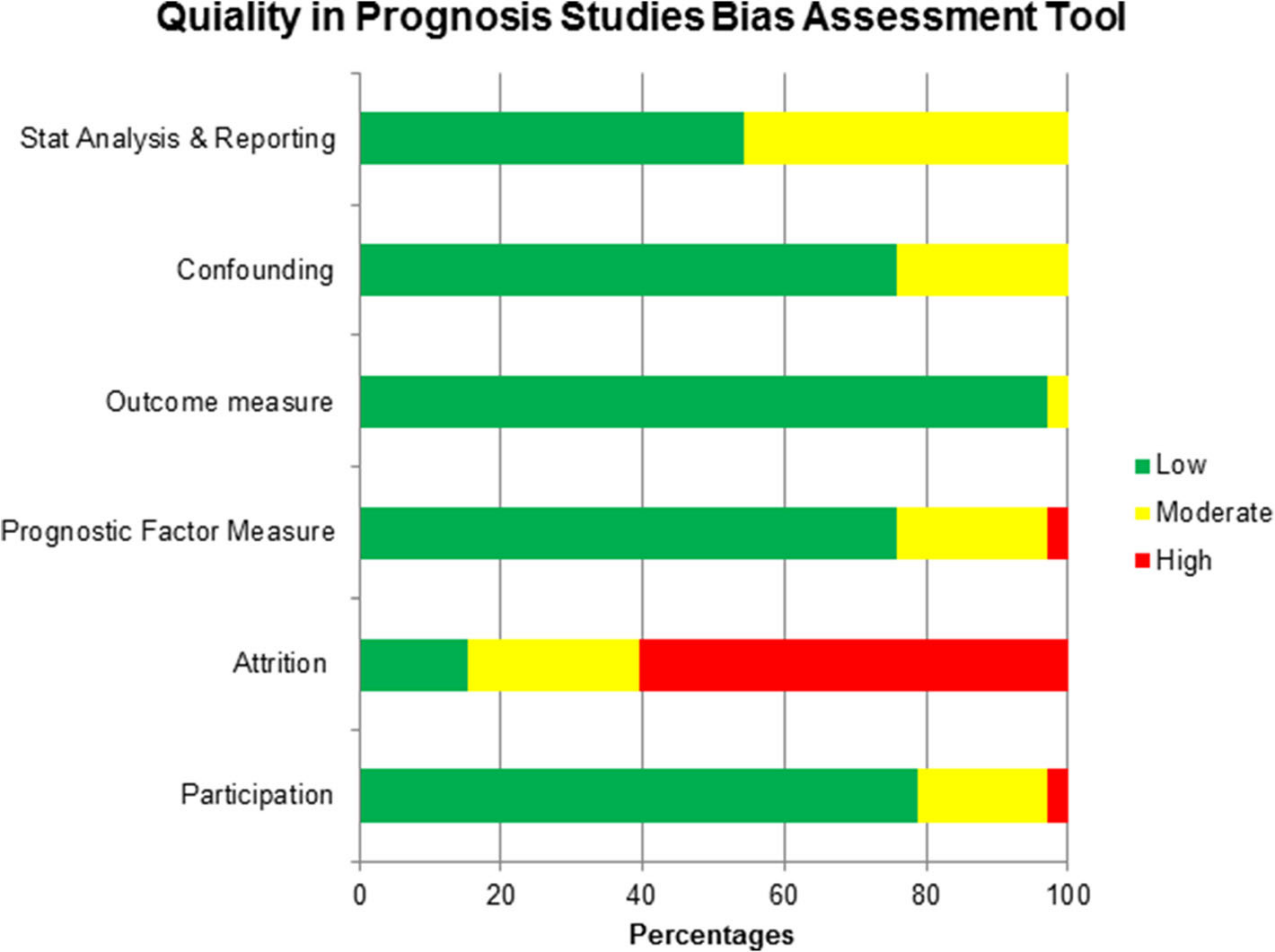
Bias assessment of included studies using the Quality in Prognosis Studies tool

The included studies were usually at predominantly low risk of bias with regard to study participation, measurement of prognostic factors, outcome measure and confounding factors. There was low to moderate risk of bias for statistical analysis because several studies selectively reported results and/ were not clear about the process of model building. The majority of studies were at high risk of attrition bias because there were notable amount of missing data and/ the number of participants’ loss to follow-up was not accounted for. Figure 2 shows the overall bias assessment.

### The types and calculation of Framingham risk score

Eleven studies (31.4%) adopted FRS 1998 [5, 39, 44, 46, 48, 52, 53, 59, 62, 66, 67], 13 studies (37.1%) adopted FRS 2002 [13, 40–43, 47, 49, 50, 54, 58, 63, 65, 68] and 3 studies adopted FRS 2008 [51, 56, 60]. Three studies used both FRS 1998 and 2002 [4, 9, 14]. Two studies did not specify the iteration of FRS used [55, 57]. According to previously published criteria [15], additions, deletions and modifications of risk factors are shown in Table 1. Six studies (17.1%) did not provide any mean estimate or a breakdown of different categories of FRS [41, 51, 55, 58, 67, 69]. The median number of items altered was 3. Using that as the threshold, twelve studies (34.3%) had major alterations [4, 9, 39, 41–44, 46, 51, 59, 67, 68] and 23 studies (65.7%) had minor alterations [5, 13, 14, 40, 45, 47–50, 52–58, 60–66]. Five of 23 studies that had minor alterations did not have any components of FRS altered [49, 55, 56, 60, 66]. Of those 5 studies, two studies did not provide any information about the components of FRS [56, 60] and were given the benefit of the doubt, however, findings should be interpreted with caution.

**Table 1:** Alteration of the risk factors used for the calculation of Framingham Risk Score in 35 eligible studies compared to the Framingham Risk Score 1998, 2002 and 2008

	No. of Studies (*n* = 35)		
	Ordinal outcomes		
Items of Alteration (*n* = 18)	Yes	No	Unclear
Addition			
Item 1. Antihypertensive	16	15	4
Item 2. Weight related measures, e.g. BMI	0	35	0
Item 3. Race/ethnic groups	2	33	0
Item 4. Triglycerides	2	33	0
Item 5. Alcohol	0	35	0
Item 6. Previous cardiovascular disease	1	34	0
Item 7. Others (family history, PVD & stroke)	5	30	0
Deletion			
Item 8. Diastolic blood pressure	9	21	5
Item 9. ^a^Diabetes	0	19	0
Item 10. HDL cholesterol	4	28	3
Modification			
Blood pressure			
Item 11. Systolic blood pressure	3	23	9
Item 12. History of hypertension/self-reported hypertension	7	23	5
Item 13. Other blood pressure definition modification	3	31	1
Lipid levels			
Item 14. History of hyperlipidaemia/ self-reported hyperlipidaemia	14	14	7
Diabetes			
Item 15. ^a^Fasting glucose > 126 mg/dL or 7.8 mmol/L	0	19	0
Item 16. ^a^Self-reported diabetes/ use diabetic medication	3	10	6
Smoking			
Item 17. Pack years of smoking	0	34	1
Item 18. Use of ex-smoker category	11	23	1

*Abbreviations: BMI* body mass index, *HDL* high density lipoprotein, *PVD* peripheral vascular disease

^a^13 studies used FRS 2002 and 3 studies used FRS 2008 and diabetes related items were discounted

### Thresholds and reporting of association

Odds ratio, relative risk, c-index and hazard ratio were used to indicate association of the imaging biomarker with outcomes. There was selective reporting of subgroups and *p* values among the reported subgroups (Table 2). The reference groups of investigations were not consistent. In CTCA, the reference group could either be no disease or non-obstructive disease. In both TACS and CACS, the reference group was not always a score of zero. The Additional file 1 displays the details regarding the thresholds of different types of investigation. The cut points that define respective categories were also variable.

**Table 2:** Selective reporting of association

	Reference groups, *n*	Subgroups, *n*	Reported subgroups, *n* (%)	Missing *p*-value, *n* (%)
All effect sizes	92	381	328 (86)	85 (26)
OR	4	27	27 (100)	9(33)
RR	8	37	37 (100)	18 (49)
C-index	0	22	22 (100)	0 (0)
HR	80	295	242 (82)	58 (24)

*Abbreviations OR* odds ratio, *RR* relative risk, *c-index* index of concordance, *HR* hazard ratio

### Intended population for Framingham risk score

Four studies (11.4%) had an exclusively Caucasian population [5, 13, 52, 53]. Eleven studies (31.4%) had more than 10% non-Caucasian population [41, 48–51, 54, 57, 58, 61, 66, 68]. Twenty two studies (62.9%) had not recorded ethnicity as a variable [4, 9, 14, 40, 42–47, 52, 53, 55, 56, 58–60, 62–65, 67]. Five studies (14.3%) had documented CHD at baseline [45, 57, 63, 65, 67]. Considering all the information, only 4 studies (11.4%) were identified as similar to the original Framingham population [5, 13, 52, 53].

### Documentation of regression, discrimination & AUC analysis

**Fig. 3:**
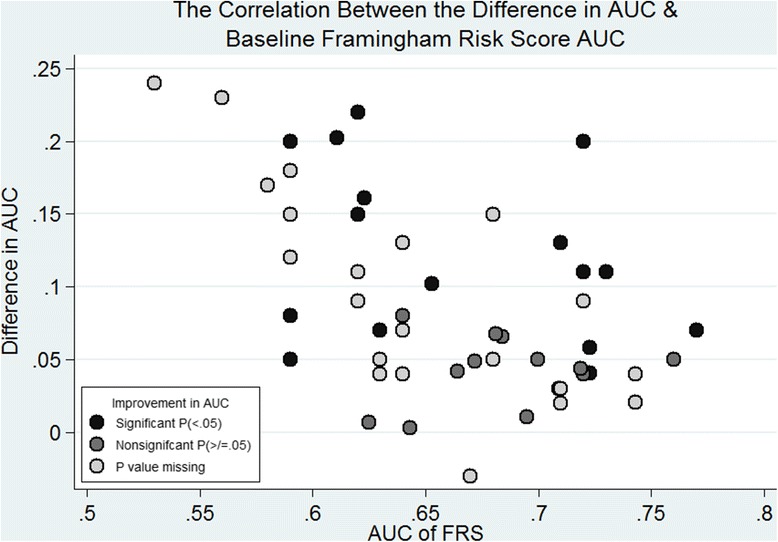
The correlation between difference in AUC and baseline Framingham Risk Score AUC

Of the 35 studies, the majority appropriately reported multivariable regression (74.3%). Thirty three studies reported AUC estimates for both FRS alone and the FRS with additional CT biomarkers with data on 76 such pairs of data. Appropriate documentation of AUC was not common practice (36.4%). The method used to compare receiver operating characteristics curves was not always described (39.4%). Only eight studies reported calibration (22.9%) [4, 48, 51–55, 68]. Table 3 shows the reporting of regression and discrimination. The AUC of FRS alone ranged from 0.53 to 0.77 (median = 0.68). The Δ AUC ranged from − 0.07 to 0.24 (median = 0.06). There was strong inverse correlation between the Δ AUC and the baseline FRS AUC (Spearman correlation coefficient, − 0.46, *p* < 0.0001). When the baseline FRS AUC performed well, the Δ AUC was relatively lower with the addition of a CT biomarker (Fig. 3).

**Table 3:** Documentation of multivariable regression, calibration, discrimination and reclassification

Part 1. Documentation of multivariable regression (*n* = 35)	No.	(%)
a. Information on whether additional predictor is significant at <.05 level	24	68.6
b. Results of a test that penalises for the inclusion of additional predictor	8	22.9
Appropriate documentation (1a or 1b)	26	74.3
Part 2. Documentation of AUC in ROC analysis (*n* = 33)		
a. Described method used to compare ROC curves	13	39.4
b. Presented the AUC values with and without the additional predictor	31	93.9
c. Presented CIs of AUC values with and without additional predictor	9	27.3
d. Presented P value for comparison	26	78.8
f. Availability or enable calculation of Δ AUC CIs	30	90.9
Appropriate documentation 1 (2a and 2b and [2c or 2d])	11	33.3
Appropriate documentation 2 (2a and 2b and [2c or {2d or 2f}])	12	36.4
Part 3. Documentation of calibration (*n* = 35)		
Documentation of Hosmer-Lemeshaw test (*n* = 7) or Schoenfeld residuals (*n* = 1)	8	22.9
Part 4. Documentation of reclassification analysis (*n* = 35)		
Report using table or text		
Not reported	19	54.3
Partial	5	14.3
Complete	11	31.4
Standard of reporting of reclassification analysis (*n* = 16)		
a. Use of standard categories of risk	11	68.8
b. Justified use of other categories of risk	15	93.8
c. Reported the number of patients changing categories	9	56.3
Appropriate documentation ([4a or 4b] and 4c)	9	56.3
Inadequate	7	43.8
Part 5. Documentation of NRI (*n* = 23)		
Type of NRIs		
Continuous/ category-free NRI	4	17.4
Categorical NRI	16	69.6
Reported both continuous & categorical NRIs	1	4.3
Reported relative NRI	1	4.3
Unclear	3	13.0
Standard of reporting of categorical NRI (*n* = 16)		
a. Report censor handling	5	31.3
b. No extrapolation	7	43.8
c. Categorical NRI reference available	14	87.5
d. Justification of risk categories	14	87.5
e. Report NRI components	5	31.3
f. Availability of reclassification table showing event and non-event	8	50.0
g. Reclassification table enables the calculation of NRI components	7	43.8
h. Combined NRI reported as a sum not a percentage	8	50.0
i. The proportion of correctly reclassified subjects available	7	43.8
j. Reported NRI not used to construct strong summary	5	31.3
Adequate reporting of categorical NRI (> 5 items listed 5a–j)^a^	11	68.8

^a^The threshold is the median number of items reported in a skewed sample

Table 4 shows the median AUC values and the Δ AUC when the data was classified according to features of design and analysis [15]. The baseline FRS AUC performs better with minor alterations compared with those with major alterations of the Framingham model (*p* = 0.0006). The improvement in AUC was greater in those with major alterations of the Framingham model (*p* = 0.015). Other factors that significantly affected the performance of AUC included the exploration of data analysis, reporting of calibration and validation, multivariable and AUC documentation (all *p* < 0.05). The types of incremental value reported were associated with a difference in AUC performance, but only significant when a threshold of 2 was chosen. In the sample population, measurement of CHD as an outcome or whether the population was similar to the original Framingham cohort did not significantly alter the AUC performance.

**Table 4:** Median AUC values and ΔAUC according to different aspects of design and analysis

	No.	AUC FRS (median)	IQR	*P* value	No.	AUC FRS + CT (median)	IQR	*P* value	No.	Δ AUC (median)	IQR	*P* value
1. Alteration of Framingham model												
Major	31	0.64	0.62–0.68		31	0.74	0.71–0.77		30	0.07	0.05–0.15	
Minor	42	0.7	0.64–0.74	0.0006	45	0.76	0.68–0.79	0.7271	46	0.05	0.02–0.09	0.015
2. Coronary heart disease measured												
Yes	58	0.68	0.62–0.72		61	0.75	0.71–0.78		61	0.06	0.04–0.11	
No	15	0.66	0.64–0.68	0.5208	15	0.72	0.68–0.75	0.2452	15	0.05	0.04–0.08	0.5393
3. Explore analysis model												
Yes	13	0.75	0.72–0.76		16	0.77	0.71–0.80		16	0.05	0.03–0.06	
No	60	0.65	0.62–0.71	< 0.0001	60	0.74	0.71–0.78	0.4559	60	0.07	0.04–0.13	0.0274
4. Population as intended for Framingham												
Yes	45	0.68	0.64–0.72		48	0.75	0.71–0.77		48	0.06	0.04–0.11	
No	28	0.64	0.63–0.71	0.1841	28	0.74	0.68–0.78	0.5901	28	0.05	0.04–0.12	0.8546
5. Calibration reporting												
Yes	19	0.69	0.64–0.72		19	0.74	0.67–0.77		19	0.04	0.01–0.06	
No	54	0.67	0.62–0.72	0.1427	57	0.75	0.71–0.78	0.1465	57	0.07	0.05–0.12	0.0007
6. Validation reporting												
Yes	22	0.65	0.62–0.70		22	0.74	0.71–0.76		22	0.08	0.05–0.13	
No	51	0.68	0.36–0.74	0.0433	54	0.76	0.68–0.78	0.7267	54	0.06	0.04–0.09	0.1231
7. Multivariable documentation												
Adequate	52	0.64	0.62–0.71		52	0.74	0.71–0.78		52	0.07	0.04–0.13	
Inadequate	21	0.72	0.68–0.75	0.003	24	0.76	0.69–0.78	0.6588	24	0.05	0.03–0.08	0.1002
8. AUC documentation												
Adequate	28	0.72	0.64–0.75		28	0.77	0.69–0.80		28	0.05	0.01–0.08	
Inadequate	45	0.66	0.62–0.70	0.0018	48	0.74	0.71–0.77	0.3431	48	0.07	0.05–0.13	0.016
9. Reclassification analysis documentation 1												
Adequate (reference)	14	0.69	0.62–0.72		17	0.76	0.74–0.78		17	0.06	0.05–0.11	
Inadequate or not reported	59	0.67	0.63–0.72	0.3924	59	0.74	0.70–0.74	0.2539	59	0.06	0.03–0.11	0.2032
Inadequate	17	0.64	0.63–0.67	0.095	17	0.73	0.70–0.76	0.1678	17	0.07	0.05–0.11	0.9035
Not reported	42	0.68	0.63–0.72	0.7189	42	0.74	0.71–0.78	0.3885	42	0.05	0.02–0.11	0.0772
10. Reclassification analysis documentation 2												
Inadequate	17	0.64	0.63–0.67		17	0.73	0.70–0.76		17	0.07	0.05–0.11	
Not reported	42	0.68	0.63–0.72	0.0443	42	0.74	0.71–0.78	0.6452	42	0.05	0.02–0.11	0.0877
11. Types of incremental value threshold												
> 2	40	0.68	0.63–0.72		43	0.74	0.68–0.76		43	0.05	0.03–0.08	
< 2	33	0.67	0.62–0.75	0.731	33	0.77	0.73–0.83	0.0013	33	0.08	0.05–0.15	0.0034
12. Types of incremental value threshold 2												
> 3	12	0.72	0.69–0.74		12	0.76	0.74–0.78		12	0.05	0.04–0.07	
< 3	61	0.66	0.62–0.71	0.0158	64	0.74	0.69–0.78	0.2884	64	0.06	0.04–0.11	0.192

*AUC* area under the operating curve, *ΔAUC* difference in AUC, *CT CT* biomarkers, *FRS* Framingham model, *IQR* interquartile range

*P* values generated using Wilcoxan ranksum test

### Documentation of reclassification and NRI analysis

Twenty three studies reported NRI estimates and all had at least 2 cut-offs, with those that had 3 cut-offs making up the biggest NRIs [59, 67]. The number of thresholds influenced the value of NRI [70]. The most commonly used type of NRI was categorical NRI (69.6%). The studies that reported calibration were the same as those documented in the last section. Table 3 shows the reporting of reclassification analysis and NRI. Complete reporting of reclassification analysis using reclassification table or text was not common practice (31.4%). When reclassification analysis was done, only half was considered appropriate (56.3%). The actual number of patients being up or down classified to a different risk group was not documented. In conjunction with the documentation of NRI, the proportion of subjects being correctly reclassified was not always available (43.8%). Most studies subjectively drew strong conclusions from the NRI calculated (68.7%). The individual components of events and non-events and also their respective NRI components were not always available (at least 43.8%). Fifteen studies reported categorical NRI with 46 data points. The values of categorical combined NRI ranged from − 0.083 to 0.785 (median = 0.249). None of the aspects of reporting [37] was significantly related to the difference in the values of categorical combined NRI (Table 5).

**Table 5:** Median NRI values according to different aspects of design and analysis

	No.	NRI (median)	IQR	*P* value
1. Reporting of censor handling				
Yes	13	0.18	0.14–0.43	
No	33	0.26	0.19–0.35	0.4869
2. No extrapolation				
Yes	20	0.28	0.14–0.49	
No	26	0.23	0.16–0.34	0.4186
3. Category NRI reference^a^				
Quoted	38	0.25	0.18–0.39	
Not quoted	8	0.18	0.00–0.38	0.1922
4. Justification of NRI categories^a^				
Yes	40	0.25	0.15–0.41	
No	6	0.21	0.18–0.29	0.7691
5. Reporting NRI components				
Yes	22	0.24	0.13–0.34	
No	24	0.28	0.19–0.48	0.1907
6. Reclassification table showing the number of events and non-events				
Yes	32	0.27	0.18–0.37	
No	14	0.21	0.14–0.43	0.3396
7. The availability of reclassification table with sufficient information to enable the calculation of event and non-event NRI				
Yes	30	0.25	0.18–0.35	
No	16	0.23	0.14–0.48	0.9265
8. Describing the combined NRI as sum not a percentage				
Yes	25	0.28	0.14–0.47	
No	21	0.22	0.18–0.30	0.256
9. Provide any indication of the proportion of correctly reclassified				
Yes	15	0.35	0.14–0.53	
No	31	0.23	0.16–0.34	0.0916
10. Strong conclusion based on the reporting of NRI				
No	20	0.29	0.19–0.48	No
Yes	26	0.23	0.13–0.34	0.08
11. NRI adequate documented				
Adequate	39	0.25	0.14–0.39	
Inadequate	7	0.24	0.20–0.49	0.7949
12. Types of incremental value				
> 3	18	0.28	0.2–0.44	
< 3	28	0.23	0.13–0.37	0.2464

*Abbreviations: IQR* interquartile range, *NRI* net reclassification index

*P* values generated using Wilcoxon ranksum test

^a^One group has less then 10 studies and therefore the comparison is limited

## Discussion

The majority of studies claimed improved discrimination and reclassification of the outlined CT biomarkers over the established Framingham model. For association, hazard ratios were commonly used but the variation in reporting practice hindered evidence synthesis. Although all studies used a similar baseline model for AUC analysis, the performance of FRS varied. There was a clear negative correlation between improved discrimination and baseline performance of FRS. In contrast, despite the poor reporting, there was no difference in the magnitude of categorical NRI between adequate and inadequate reporting practice.

Selective reporting of association is known and remains an issue [16]. We found that non-standardisation of thresholds and different reference groups across studies prohibit future meta-analysis. Here we substantiate this with 3 included studies. Chow et al. used non-obstructive coronary disease as the reference group for 3 different composite outcomes [65]. The same author then used no coronary artery disease as the reference group in the CONFIRM cohort [63]. In [66], we estimated hazard ratios using Kaplan-Meier curve [34]. When non-obstructive coronary disease is the reference group, the estimated association of obstructive disease with composite outcome is smaller (HR = 2.03, 95% CI 1.47–2.79), compared with when no coronary disease/normal is the reference, the estimated association is bigger (HR = 3.24, 95% CI 2.28–4.63) [66].

NRI records the transformation in predicted risk that changes from one category to another category after the introduction of an additional test. However, it is only meaningful when the information about risk thresholds is available. The change in predicted risk could be correct or incorrect. In this population, the concept that the subject could be wrongly reclassified with an additional test was not clearly outlined. The combined NRI could have been driven by predominantly event NRI, leading to overestimation. This could have been clarified by reporting of the components of NRI but this was not standard practice, despite recommendation [17]. Deriving from concern about miscalibration, another recommendation was the regular reporting of calibration [26, 71, 72], with graphical plot being the best assessment [73]. Calibration, however, was regularly overlooked. To counteract the issues with missing data, we have met solutions such as Weibull extrapolation [5, 44, 47] and adjustment of risk cut-offs by the ratio of actual follow up. These strategies translate to a fact that a significant proportion of the included studies used non-standardised risk categories but almost all managed to justify. A more definitive solution would be a move towards decision curve analysis [74]. Only 1 study provided information to allow adjustment using Kaplan-Meier estimates [54]. This is on a background of insufficient reporting on the handling of censoring. This adjustment should receive more attention, especially when censoring happened early on during follow-up [75]. There is currently no consensus on what is a large enough NRI. Overall, considering the uncertainty in NRI [17] and the small values of NRI, one should not draw strong conclusions from the use of NRI alone. Given the popularity of NRI in cardiovascular research [26], a framework of reporting NRI should be followed, for example in [37].

Discrimination as measured by AUC analysis is an established method of measuring incremental value [76]. It was reported in almost all the studies but adequate documentation was not common practice. In our study, reporting of calibration, validation and AUC documentation all influenced the values of AUC. The baseline Framingham model is an established score even considering its various iterations [1]. Big improvements in AUC was seen in cases where the baseline model performed badly. This echoes previous findings [15] but in a more defined population. As eluded to in [15], this phenomenon is similar to when a new drug is only effective when compared to an ineffective comparator drug [77]. In addition to the above, inadequate reporting practices were associated with inflated estimates.

Our investigation on AUC [15] and NRI [17] were not empirical and can only serve as an update in a different population. The assessment on thresholds was minimal compared with previous investigation [17]. The harm of imaging using CT (radiation burden) was not explored. The focus was solely on the potential benefit. Studies that indicated association or only had a reclassification table could be excluded because we focused on studies that had at least 1 summary estimate that indicated incremental value. We were unable to assess publication bias where articles showed no or worsening prediction if these studies remain unpublished. The calculation of model coefficients can significantly impact the baseline AUC. There are different ways of calculating the model coefficients (re-estimation for the new population, using published coefficients or a point based model) but these were not explored. The difference between using CHD or CVD as an outcome was investigated, however, the justification and transparency of reporting outcomes was not examined.

Association on its own is insufficient to substantiate incremental value [78] and large values are infrequent in biomarker research [79]. AUC analysis is seen as a good starting point and reclassification should follow rather than replace AUC analysis [76]. However, AUC analysis is not without fault [79]. Transparent reporting of NRI should be compulsory, for example use of a reclassification table (38), and readers should be aware of the controversies surrounding NRI [80]. The co-existence of a lack of increase in AUC and a positive NRI should alarm readers [81]. In general, the reporting in prognosis studies needs to be more robust [82–85]. Data should be made available for individual patient data meta-analysis.

## Conclusion

Inconsistent thresholds, reference groups and selective reporting prohibit future evidence synthesis of associations. Inadequate documentation of discrimination, calibration and validation are widespread. The variable baseline performance and other aspects of reporting discrimination inflate potential incremental values. Reporting of reclassification is also insufficient but significant differences between adequate and inadequate reporting practice have not been identified.

## Additional file

Additional file 1: Threshold information of coronary and thoracic calcium scores, and computed tomographic coronary angiogram. (DOCX 18 kb)

## Abbreviations

AUC: Area under the receiver operating characteristics curve
CACS: Coronary calcium score
CHD: Coronary heart disease
CONFIRM: CoroNary CT Angiography Evaluation For Clinical Outcomes: An InterRnational Multicenter Registry (CONFIRM)
CT: Computed tomography
CTCA: Computed tomographic coronary angiogram
FRS: Framingham Risk Score
IDI: Integrated discrimination improvement
NRI: Net reclassification index
PRISMA: Preferred Reporting Items of Systematic Review and Meta-analysis
rIDI: Relative integrated discrimination improvement
TACS: Thoracic aorta calcium score
Δ AUC: Difference in area under the receiver operating characteristics curve

## References

[1] Damen JAAG, Hooft L, Schuit E, Debray TPA, Collins GS, Tzoulaki I, et al. Prediction models for cardiovascular disease risk in the general population: systematic review. BMJ. 2016;353:i2416. 10.1136/bmj.i2416 PMC4868251 27184143

[2] Bouwmeester W, Zuithoff NP, Mallett S, Geerlings MI, Vergouwe Y, Steyerberg EW (2012). Reporting and methods in clinical prediction research: a systematic review. PLoS Med.

[3] Wilson PW, D'Agostino RB, Levy D, Belanger AM, Silbershatz H, Kannel WB (1998). Prediction of coronary heart disease using risk factor categories. Circulation.

[4] Erbel R, Mohlenkamp S, Moebus S, Schmermund A, Lehmann N, Stang A (2010). Coronary risk stratification, discrimination, and reclassification improvement based on quantification of subclinical coronary atherosclerosis: the Heinz Nixdorf recall study. J Am Coll Cardiol.

[5] Kavousi M, Elias-Smale S, Rutten JH, Leening MJ, Vliegenthart R, Verwoert GC (2012). Evaluation of newer risk markers for coronary heart disease risk classification: a cohort study. Ann Intern Med.

[6] Moons KGM, Royston P, Vergouwe Y, Grobbee DE, Altman DG. Prognosis and prognostic research: what, why, and how? BMJ. 2009;338 10.1136/bmj.b375 19237405

[7] Hlatky MA, Greenland P, Arnett DK, Ballantyne CM, Criqui MH, Elkind MS (2009). Criteria for evaluation of novel markers of cardiovascular risk: a scientific statement from the American Heart Association. Circulation.

[8] O'Connor JP, Aboagye EO, Adams JE, Aerts HJ, Barrington SF, Beer AJ (2017). Imaging biomarker roadmap for cancer studies. Nat Rev Clin Oncol.

[9] Valenti V, B OH, Heo R, Cho I, Schulman-Marcus J, Gransar H, et al. A 15-year warranty period for asymptomatic individuals without coronary artery calcium: a prospective follow-up of 9,715 individuals. JACC Cardiovasc Imaging 2015;8(8):900–909. 10.1016/j.jcmg.2015.01.025 PMC4537357 26189116

[10] Oudkerk M, Stillman AE, Halliburton SS, Kalender WA, Möhlenkamp S, McCollough CH (2008). Coronary artery calcium screening: current status and recommendations from the European Society of Cardiac Radiology and North American Society for cardiovascular imaging. Int J Cardiovasc Imaging.

[11] Ioannidis JP, Tzoulaki I (2010). What makes a good predictor?: the evidence applied to coronary artery calcium score. JAMA.

[12] Agatston AS, Janowitz WR, Hildner FJ, Zusmer NR, Viamonte M, Detrano R (1990). Quantification of coronary artery calcium using ultrafast computed tomography. J Am Coll Cardiol.

[13] Wong ND, Gransar H, Shaw L, Polk D, Moon JH, Miranda-Peats R (2009). Thoracic aortic calcium versus coronary artery calcium for the prediction of coronary heart disease and cardiovascular disease events. JACC-Cardiovasc Imag..

[14] Hadamitzky M, Achenbach S, Al-Mallah M, Berman D, Budoff M, Cademartiri F (2013). Optimized prognostic score for coronary computed tomographic angiography: results from the CONFIRM registry (COronary CT angiography EvaluatioN for clinical outcomes: an InteRnational multicenter registry). J Am Coll Cardiol.

[15] Tzoulaki I, Liberopoulos G, Ioannidis JP (2009). Assessment of claims of improved prediction beyond the Framingham risk score. JAMA.

[16] Kyzas PA, Loizou KT, Ioannidis JP (2005). Selective reporting biases in cancer prognostic factor studies. J Natl Cancer Inst.

[17] Tzoulaki I, Liberopoulos G, Ioannidis JP (2011). Use of reclassification for assessment of improved prediction: an empirical evaluation. Int J Epidemiol.

[18] Liberati A, Altman DG, Tetzlaff J, Mulrow C, Gotzsche PC, Ioannidis JP (2009). The PRISMA statement for reporting systematic reviews and meta-analyses of studies that evaluate healthcare interventions: explanation and elaboration. BMJ.

[19] Pang CL, Peters J, Hyde C, Roobottom C. The added value of computed tomography coronary angiogram in predicting future cardiovascular events in a low risk population: comparison with Framingham Risk Score. PROSPERO: International prospective register for systematic reviews. 2015:CRD42015023795.

[20] British Library e-theses online service. http://ethos.bl.uk/Home.do. Accessed Sept 03, 2015.

[21] System for Information on Grey Literature in Europe. http://www.opengrey.eu/. Accessed Sept 03, 2015.

[22] DeLong ER, DeLong DM, Clarke-Pearson DL (1988). Comparing the areas under two or more correlated receiver operating characteristic curves: a nonparametric approach. Biometrics.

[23] Kerr KF, Wang Z, Janes H, McClelland RL, Psaty BM, Pepe MS. Net Reclassification indices for evaluating risk-prediction instruments: a critical review. Epidemiology (Cambridge, Mass). 2014;25(1):114–121. 10.1097/EDE.0000000000000018 PMC3918180 24240655

[24] Pencina MJ, D'Agostino RB, Demler OV (2012). Novel metrics for evaluating improvement in discrimination: net reclassification and integrated discrimination improvement for normal variables and nested models. Stat Med.

[25] Pencina MJ, D'Agostino RB, Steyerberg EW (2011). Extensions of net reclassification improvement calculations to measure usefulness of new biomarkers. Stat Med.

[26] Pencina MJ, D'Agostino RB, D'Agostino RB, Vasan RS (2008). Evaluating the added predictive ability of a new marker: from area under the ROC curve to reclassification and beyond. Stat Med.

[27] Pencina MJ, D'Agostino RB, D'Agostino RB, Vasan RS (2008). Comments on ‘integrated discrimination and net reclassification improvements—practical advice’. Stat Med.

[28] Ferreira-González I, Permanyer-Miralda G, Domingo-Salvany A, Busse JW, Heels-Ansdell D, Montori VM (2007). Problems with use of composite end points in cardiovascular trials: systematic review of randomised controlled trials. BMJ.

[29] Ciani O, Buyse M, Garside R, Pavey T, Stein K, Sterne JAC, et al. Comparison of treatment effect sizes associated with surrogate and final patient relevant outcomes in randomised controlled trials: meta-epidemiological study. BMJ. 2013;346:f457. 10.1136/bmj.f457 PMC3558411 23360719

[30] Third Report of the National Cholesterol Education Program (NCEP) Expert Panel on Detection, Evaluation, and Treatment of High Blood Cholesterol in Adults (Adult Treatment Panel III) Final Report. Circulation. 2002;106(25):3143. 12485966

[31] D’Agostino RB, Vasan RS, Pencina MJ, Wolf PA, Cobain M, Massaro JM (2008). General cardiovascular risk profile for use in primary care: the Framingham heart study. Circulation.

[32] Hanley JA, McNeil BJ (1983). A method of comparing the areas under receiver operating characteristic curves derived from the same cases. Radiology.

[33] Altman DG, Bland JM. How to obtain the P value from a confidence interval. BMJ. 2011;343:d2304. 10.1136/bmj.d2304 22803193

[34] Guyot P, Ades AE, Ouwens MJNM, Welton NJ (2012). Enhanced secondary analysis of survival data: reconstructing the data from published Kaplan-Meier survival curves. BMC Med Res Methodol.

[35] Leeflang MM, Moons KG, Reitsma JB, Zwinderman AH (2008). Bias in sensitivity and specificity caused by data-driven selection of optimal cutoff values: mechanisms, magnitude, and solutions. Clin Chem.

[36] Pencina MJ, D'Agostino RB, Pencina KM, Janssens AC, Greenland P (2012). Interpreting incremental value of markers added to risk prediction models. Am J Epidemiol.

[37] Leening MJG, Vedder MM, Witteman JCM, Pencina MJ, Steyerberg EW (2014). Net reclassification improvement: computation, interpretation, and ControversiesA literature review and Clinician's guide. Ann Intern Med.

[38] Hayden JA, van der Windt DA, Cartwright JL, Cote P, Bombardier C (2013). Assessing bias in studies of prognostic factors. Ann Intern Med.

[39] Lau KK, Wong YK, Chan YH, Yiu KH, Teo KC, Li LS, et al. Prognostic implications of surrogate markers of atherosclerosis in low to intermediate risk patients with Type 2 Diabetes. Cardiovasc Diabetol. 2012;11(101). 10.1186/1475-2840-11-101 PMC3444371 22900680

[40] Ahmadi N, Hajsadeghi F, Blumenthal RS, Budoff MJ, Stone GW, Ebrahimi R (2011). Mortality in individuals without known coronary artery disease but with discordance between the Framingham risk score and coronary artery calcium. Am J Cardiol.

[41] Budoff MJ, Shaw LJ, Liu ST, Weinstein SR, Mosler TP, Tseng PH (2007). Long-term prognosis associated with coronary calcification: observations from a registry of 25,253 patients. J Am Coll Cardiol.

[42] Raggi P, Shaw LJ, Berman DS, Callister TQ (2004). Gender-based differences in the prognostic value of coronary calcification. J Women's Health.

[43] Chang SM, Nabi F, Xu J, Pratt CM, Mahmarian AC, Frias ME (2015). Value of CACS compared with ETT and myocardial perfusion imaging for predicting long-term cardiac outcome in asymptomatic and symptomatic patients at low risk for coronary disease clinical implications in a multimodality imaging world. JACC-Cardiovasc Imag.

[44] Elias-Smale SE, Proenca RV, Koller MT, Kavousi M, van Rooij FJ, Hunink MG (2010). Coronary calcium score improves classification of coronary heart disease risk in the elderly: the Rotterdam study. J Am Coll Cardiol.

[45] Forouzandeh F, Chang SM, Muhyieddeen K, Zaid RR, Trevino AR, Xu J (2013). Does quantifying epicardial and intrathoracic fat with noncontrast computed tomography improve risk stratification beyond calcium scoring alone?. Circ Cardiovasc Imag..

[46] Hadamitzky M, Meyer T, Hein F, Bischoff B, Martinoff S, Schomig A (2010). Prognostic value of coronary computed tomographic angiography in asymptomatic patients. Am J Cardiol.

[47] Elias-Smale SE, Wieberdink RG, Odink AE, Hofman A, Hunink MG, Koudstaal PJ (2011). Burden of atherosclerosis improves the prediction of coronary heart disease but not cerebrovascular events: the Rotterdam study. Eur Heart J.

[48] Yeboah J, Carr JJ, Terry JG, Ding J, Zeb I, Liu S (2014). Computed tomography-derived cardiovascular risk markers, incident cardiovascular events, and all-cause mortality in nondiabetics: the multi-ethnic study of atherosclerosis. Eur J Prev Cardiol.

[49] Greenland P, LaBree L, Azen SP, Doherty TM, Detrano RC (2004). Coronary artery calcium score combined with Framingham score for risk prediction in asymptomatic individuals.[erratum appears in JAMA. 2004 Feb 4;291(5):563]. JAMA.

[50] Yeboah J, McClelland RL, Polonsky TS, Burke GL, Sibley CT, O'Leary D (2012). Comparison of novel risk markers for improvement in cardiovascular risk assessment in intermediate-risk individuals. JAMA.

[51] Matsushita K, Sang YY, Ballew SH, Shlipak M, Katz R, Rosas SE (2015). Subclinical atherosclerosis measures for cardiovascular prediction in CKD. J Am Soc Nephrol.

[52] Mohlenkamp S, Lehmann N, Greenland P, Moebus S, Kalsch H, Schmermund A (2011). Coronary artery calcium score improves cardiovascular risk prediction in persons without indication for statin therapy. Atherosclerosis.

[53] Mohlenkamp S, Lehmann N, Moebus S, Schmermund A, Dragano N, Stang A (2011). Quantification of coronary atherosclerosis and inflammation to predict coronary events and all-cause mortality. J Am Coll Cardiol.

[54] Polonsky TS, McClelland RL, Jorgensen NW, Bild DE, Burke GL, Guerci AD (2010). Coronary artery calcium score and risk classification for coronary heart disease prediction. JAMA.

[55] Raggi P, Cooil B, Callister TQ (2001). Use of electron beam tomography data to develop models for prediction of hard coronary events. Am Heart J.

[56] Rana JS, Gransar H, Wong ND, Shaw L, Pencina M, Nasir K (2012). Comparative value of coronary artery calcium and multiple blood biomarkers for prognostication of cardiovascular events. Am J Cardiol.

[57] Agarwal S, Cox AJ, Herrington DM, Jorgensen NW, Xu J, Freedman BI (2013). Coronary calcium score predicts cardiovascular mortality in diabetes: diabetes heart study. Diabetes Care.

[58] Arad Y, Goodman KJ, Roth M, Newstein D, Guerci AD (2005). Coronary calcification, coronary disease risk factors, C-reactive protein, and atherosclerotic cardiovascular disease events: the St. Francis heart study. J Am Coll Cardiol.

[59] Cho I, Chang HJ, Sung JM, Pencina MJ, Lin FY, Dunning AM (2012). Coronary computed tomographic angiography and risk of all-cause mortality and nonfatal myocardial infarction in subjects without chest pain syndrome from the CONFIRM registry (coronary CT angiography evaluation for clinical outcomes: an international multicenter registry). Circulation.

[60] Versteylen MO, Kietselaer BL, Dagnelie PC, Joosen IA, Dedic A, Raaijmakers RH (2013). Additive value of Semiautomated quantification of coronary artery disease using cardiac computed tomographic angiography to predict future acute coronary syndrome. J Am Coll Cardiol.

[61] Gibson AO, Blaha MJ, Arnan MK, Sacco RL, Szklo M, Herrington DM (2014). Coronary artery calcium and incident cerebrovascular events in an asymptomatic cohort the MESA study. JACC-Cardiovasc Imag..

[62] Hermann DM, Gronewold J, Lehmann N, Moebus S, Jockel KH, Bauer M (2013). Coronary artery calcification is an independent stroke predictor in the general population. Stroke.

[63] Chow BJ, Small G, Yam Y, Chen L, Achenbach S, Al-Mallah M (2011). Incremental prognostic value of cardiac computed tomography in coronary artery disease using CONFIRM: COroNary computed tomography angiography evaluation for clinical outcomes: an InteRnational multicenter registry. Circ Cardiovasc Imag.

[64] Lin FY, Shaw LJ, Dunning AM, LaBounty TM, Choi JH, Weinsaft JW (2011). Mortality risk in symptomatic patients with nonobstructive coronary artery disease a prospective 2-center study of 2,583 patients undergoing 64-detector row coronary computed tomographic angiography. J Am Coll Cardiol.

[65] Chow BJ, Wells GA, Chen L, Yam Y, Galiwango P, Abraham A (2010). Prognostic value of 64-slice cardiac computed tomography severity of coronary artery disease, coronary atherosclerosis, and left ventricular ejection fraction. J Am Coll Cardiol.

[66] Park HE, Chun EJ, Choi SI, Lee SP, Yoon CH, Kim HK (2013). Clinical and imaging parameters to predict cardiovascular outcome in asymptomatic subjects. Int J Cardiovasc Imag.

[67] Cho I, Chang HJ, Hartaigh BO, Shin S, Sung JM, Lin FY (2015). Incremental prognostic utility of coronary CT angiography for asymptomatic patients based upon extent and severity of coronary artery calcium: results from the COronary CT angiography EvaluatioN for clinical outcomes InteRnational multicenter (CONFIRM) study. Eur Heart J.

[68] Han D, B OH, Gransar H, Yoon JH, Kim KJ, Kim MK, et al. Incremental benefit of coronary artery calcium score above traditional risk factors for all-cause mortality in asymptomatic Korean adults. Circulation J 2015;79(11):2445–2451. 10.1253/circj.CJ-15-0651 26356835

[69] Raggi P, Gongora MC, Gopal A, Callister TQ, Budoff M, Shaw LJ (2008). Coronary artery calcium to predict all-cause mortality in elderly men and women. J Am Coll Cardiol.

[70] Muhlenbruch K, Heraclides A, Steyerberg EW, Joost HG, Boeing H, Schulze MB (2013). Assessing improvement in disease prediction using net reclassification improvement: impact of risk cut-offs and number of risk categories. Eur J Epidemiol.

[71] Pepe MS, Feng Z, Gu JW (2008). Comments on 'Evaluating the added predictive ability of a new marker: from area under the ROC curve to reclassification and beyond' by M. J. Pencina et al., statistics in medicine (DOI: 10.1002/sim.2929). Stat Med.

[72] Hilden J, Gerds TA (2014). A note on the evaluation of novel biomarkers: do not rely on integrated discrimination improvement and net reclassification index. Stat Med.

[73] Cox DR (1958). Two further applications of a model for binary regression. Biometrika.

[74] Vickers AJ, Elkin EB (2006). Decision curve analysis: a novel method for evaluating prediction models. Medical Decision Making.

[75] Steyerberg EW, Pencina MJ (2010). Reclassification calculations for persons with incomplete follow-up. Ann Intern Med.

[76] Janssens ACJW, Khoury MJ (2010). Assessment of improved prediction beyond traditional risk factors. Circ Cardiovasc Genet.

[77] Bero L, Oostvogel F, Bacchetti P, Lee K (2007). Factors associated with findings of published trials of drug–drug comparisons: why some statins appear more efficacious than others. PLoS Med.

[78] Pepe MS, Janes H, Longton G, Leisenring W, Newcomb P (2004). Limitations of the odds ratio in gauging the performance of a diagnostic, prognostic, or screening marker. Am J Epidemiol.

[79] Moons KGM (2010). Criteria for scientific evaluation of novel markers: a perspective. Clin Chem.

[80] Pepe MS, Fan J, Feng Z, Gerds T, Hilden J (2015). The net reclassification index (NRI): a misleading measure of prediction improvement even with independent test data sets. Stat Biosci.

[81] Mihaescu R, van Zitteren M, van Hoek M, Sijbrands EJ, Uitterlinden AG, Witteman JC (2010). Improvement of risk prediction by genomic profiling: reclassification measures versus the area under the receiver operating characteristic curve. Am J Epidemiol.

[82] Hemingway H, Croft P, Perel P, Hayden JA, Abrams K, Timmis A, et al. Prognosis research strategy (PROGRESS) 1: a framework for researching clinical outcomes. BMJ. 2013;346 10.1136/bmj.e5595 PMC3565687 23386360

[83] Riley RD, Hayden JA, Steyerberg EW, Moons KGM, Abrams K, Kyzas PA (2013). Prognosis research strategy (PROGRESS) 2: prognostic factor research. PLoS Med.

[84] Steyerberg EW, Moons KGM, van der Windt DA, Hayden JA, Perel P, Schroter S (2013). Prognosis research strategy (PROGRESS) 3: prognostic model research. PLoS Med.

[85] Hingorani AD, Windt DAvd, Riley RD, Abrams K, Moons KGM, Steyerberg EW, et al. Prognosis research strategy (PROGRESS) 4: Stratified medicine research. BMJ. 2013;346:e5793. 10.1136/bmj.e5793 PMC3565686 23386361

