# Supplementary material for: A methodology review on the incremental prognostic value of computed tomography biomarkers in addition to Framingham risk score in predicting cardiovascular disease: the use of association, discrimination and reclassification
Source: BMC Cardiovasc Disord. 2018 Feb 21;18:39. doi: 10.1186/s12872-018-0777-5 (PMC5822603; doi:10.1186/s12872-018-0777-5)
Supplement: Supplementary file 1 — Threshold information of coronary and thoracic calcium scores, and computed tomographic coronary angiogram. (DOCX 18 kb) [file 12872_2018_777_MOESM1_ESM.docx]

| Coronary calcium score | | | | | | |
| --- | --- | --- | --- | --- | --- | --- |
| Author | Year | Reference Group | Group 1 | Group 2 | Group 3 | Other Groups |
| Agarwal | 2013 | <10 | 10-99. | 100-299 | 300-999, >1000 | log, ordinal |
| Ahmadi | 2011 | Discorcondant low risk | Discorcondant high risk |  |  | Discorcondant high risk & 4 adjustments, CAC in discorcondant low risk |
| Arad | 2005 | 0 | 1-99. | 100-399 | >/=400 | n/a |
| Budoff | 2007 | 0, absence of vesssel >100, 3 groups from cumulative survival curve | 1-10, 11-100 | 101-399 | 400-699, 999, >1000 | 1-vessel >100, 2-vessel >100, 3-vessel >100, any vessel >100 unadjusted, any vessel >100 adjusted |
| Budoff | 2007 | 0 (3 subgroups) | 1-10, 11-100 | 101-299, 300-399 | 400-699, 700-999, >1000 | 7 other subgroups |
| Chang | 2015 | 0 | </=10, 11-100 | 101-400 | >400 | log, 3 other adjusted log |
| Cho | 2012 | 0 for 2 outcomes & 2 adjusted groups | 1-100 (for 2 outcomes) | 101-400 ((for 2 outcomes) | >400 (for 2 outcomes) | 6 adjusted groups |
| Cho | 2015 | ?</=10 | </=100 | 100-400 | 400-1000, >1000 | n/a |
| Elias-Smale | 2010 | 0 | >0 | n/a | n/a | log |
| Elias-Smale | 2011 | 1st tertitles for 2 outcomes & 3 adjustments | 2nd tertiles (for 3 adjustments) | 3rd tertiles (for 3 adjustments) | n/a | n/a |
| Erbel | 2010 | 0 for unadjusted & adjusted, 1st quartile for unadjusted & adjusted | 1-99, 2nd quartile. Both for unadjusted & adjusted. | 100-399, 3rd quartile. Both for unadjusted & adjusted. | 400-999, >/=400, >/=1000, 4th quartile. All for unadjusted & adjusted. | log unadjusted & adjusted |
| Forouzandeh | 2013 | 0 for unadjusted & 3 adjustments | 1-100 | 100-400 | >400 | >0 for unadjusted & 3 adjustments |
| Gibson | 2014 | 0 for 3 outcomes & unadjusted & adjusted | 0-100 | 100-400 | >400 | log for 3 outcomes & unadjusted & adjusted, >0 for 3 outcomes & unadjusted & adjusted |
| Greenland | 2004 | 0, 0 & FRS 0-9, 0 & FRS 10-15, 1-100 & FRS 0-9, 101-300 & FRS 0-9 | 1-100, 1-100 & FRS categories 1-4 | 101-300, 101-300 & FRS categories 1-4 | >/=301, >/=301 & FRS categories 1-4 | trend, per SD increase |
| Hermann | 2013 | ?0 | 1-99. | 100-399 | >/=400 | log (including unadjusted & 3 adjustments), men log (unadjusted & 2 adjustments), women log (unadjusted & 1 adjustment) |
| Kavousi | 2012 | n/a | n/a | n/a | n/a | log, men log, women log |
| Lau | 2012 | <40 | >40 | n/a | n/a | n/a |
| Matsushita | 2015 | non-CKD quartile 2, CKD quartile 2 | non-CKD quartile 3, CKD quartile 3 | non-CKD quartile 4, CKD quartile 4 | n/a | n/a |
| Mohlenkamp | 2011 Cor | 0 for unadjusted & adjusted | 1-99 for unadjusted & adjusted | 100-399 for unadjusted & adjusted | >/=400 for unadjusted & adjusted | n/a |
| Mohlenkamp | 2011 Quan | 0 (for 2 different outcomes, unadjusted and 2 adjustments) | 1-99 (for 2 different outcomes, unadjusted and 2 adjustments) | 100-399 (for 2 different outcomes, unadjusted and 2 adjustments) | >/=400 (for 2 different outcomes, unadjusted and 2 adjustments) | n/a |
| Park | 2013 | 0 | 0-100 | 100-400 | >400 | n/a |
| Polonsky | 2010 | n/a | n/a | n/a | n/a | log |
| Raggi | 2001 | 0 decile | >0, percentile univariate, percentile multivariate, 10th, 20th, 30th decile | 40th, 50th, 60th decile | 70th, 80th, 90th decile | log, >0 |
| Raggi | 2004 | <10 (for men & women) | 11-100 (for men & women) | 101-400 (for men & women) | 401-1000 , >1000 (for men & women) | n/a |
| Rana | 2012 | n/a | n/a | n/a | n/a | log |
| Wong | 2009 | <10 (for 3 outcomes) | 10 to 99 (for 2 outcomes) | 100-399 (for 3 outcomes) | >/=400 (for 3 outcomes) | log (for 3 outcomes) |
| Yeboah | 2009 | n/a | n/a | n/a | n/a | univariate log, multivariate log (adjusted only) |
| Yeboah | 2014 | n/a | n/a | n/a | n/a | univariate log (for 3 outcomes), multivariate log (for 3 outcomes) |
| Han | 2015 | 0 (unadjusted & adjusted) | 1-100 (unadjusted & adjusted) | 101-400 (unadjusted & adjusted) | >400 (unadjusted & adjusted) | log (unadjusted & adjusted) |
| Valenti | 2015 | 0 (undjusted & 2 adjustments) | 10-99 (undjusted & 2 adjustments) | 100-399 (undjusted & 2 adjustments) | 400-999 (undjusted & 2 adjustments), >1000 (undjusted & 2 adjustments) | >0 (undjusted & adjusted) |
| Computed tomographic coronary angiogram | | | | | | |
| Cho | 2012 | none/ non-obstructive (unadjusted & adjusted), none/ normal (unadjusted & 2 adjustments) | non-obstructive (unadjusted & 2 adjustments) | obstructive CAD (unadjusted & adjusted) | 1-vessel disease (unadjusted & 2 adjustments), 2-vessel disease (unadjusted & 2 adjustments), 3-vessel disease/ LMS (unadjusted & 2 adjustments) | n/a |
| Chow | 2011 | no CAD (3 adjustmetns) | non-obstructive (3 adjustmetns) | obstructive low-risk (3 adjustmetns) | obstructive high risk (3 adjustmetns) | CAD severity (3 adjustmetns) |
| Cho | 2015 | ? | non-obstructive | obstructive | 1-vessel disease, 2-vessel disease, 3-vessel disease/ LMS | n/a |
| Hadamitzky | 2010 | n/a | n/a | n/a | n/a | presence of CAD, most severe stenosis, number of arteries narrowed, LMS/ proximal LAD stenosis |
| Hadamitzky | 2010 | non-obstructive | obstructive | n/a | n/a | n/a |
| Hadamitzky | 2013 | n/a | no. of segments with any plaque or stenosis (c-index unadjusted & adjusted) | no. of segments with stenosis >50% (HR, c-index unadjusted & adjusted), no. of proximal segments with >50% stenosis (c-index unadjusted & adjusted) | no. of segments with stenosis >70% (HR, c-index unadjusted & adjusted) | no. of segments with non-calcified plaques, no. of segments with mixed plaques, no. of segments with calcified plaques (c-index unadjusted & adjusted), no. of segments with mixed or calcified or plaques (c-index unadjusted & adjusted), no. of proximal segments with calcified or mixed plaques (c-index unadjusted & adjusted) |
| Hadamitzky | 2013 | no. of segments with non-calcified plaques | no. of segments with any plaque or stenosis | no. of segments with stenosis >50%, no. of proximal segments with >50% stenosis | no. of segments with stenosis >70% | no. of segments with mixed plaques, no. of segments with calcified plaques , no. of segments with mixed or calcified or plaques, no. of proximal segments with calcified or mixed plaques |
| Chow | 2010 | non-obstructive CAD (for 3 outcomes) | n/a | obstructive >50% (for 3 outcomes), obstructive but not high risk (for 3 outcomes) | obstructive >70% (for 3 outcomes), obstructive high risk (for 3 outcomes) | no CAD (for 3 outcomes), CAD severity (for 3 outcomes) |
| Lin | 2011 | ? | n/a | n/a | 1-vessel disease (unadjusted & 2 adjustments), 2-vessel disease (unadjusted & 2 adjustments), 3-vessel disease (unadjusted & 2 adjustments) | 1-4 segments (unadjusted & 2 adjustments), >/=5 segments (unadjusted & 2 adjustments), non-calcified plaque (unadjusted & 2 adjustments), mixed plaque (unadjusted & 2 adjustments), calcified plaque (unadjusted & 2 adjustments) |
| Park | 2013 | 0% stenosis | 1-49% stenosis | 50-69% stenosis/ LMS 1-49% | >/=70%/ LMS 50% | degree of stenosis (unadjusted & adjusted) |
| Park | 2013 | 0% stenosis | 1-49% stenosis | 50-69% stenosis/ LMS 1-49% | >/=70%/ LMS 50% | n/a |
| Vertseylen | 2013 | n/a | n/a | n/a | n/a | n/a |
| Thoracic aortic calcium score | | | | | | |
| Elias-Smale | 2011 | 1st tertile (unadjusted & 2 adjustments) | 2nd tertile (unadjusted & 2 adjustments) | 3rd tertile (unadjusted & 2 adjustments) | n/a | n/a |
| Wong | 2009 | <10 for 3 different outcomes (hard CHD, total CHD & total CVD, for adjusted & unadjusted) | 10-99 (hard CHD, adjusted & unadjusted) | 100-399 (total CHD & total CVD, adjusted & unadjusted) | n/a | adjusted & unadjusted log for hard CHD, total CHD & total CVD |
| Yeboah | 2014 | n/a | n/a | n/a | n/a | Univariate & mulitvariate log for 3 different outcomes (incident CVD, CAD & all-cause mortality) |
| Abbreviations: CAC = coronary calcium score; CHD = coronary heart disease; CKD = chronic kidney disease; | | | | | | |
| c-index = concordance index; CVD = cardiovascular disease; FRS = Framingham Risk Score; | | | | | | |
| SD = standard deviation; HR = hazard ratio; LAD = left anterior descending; LMS = left main stem; | | | | | | |
